# Non contiguous-finished genome sequence and description of *Microbacterium gorillae* sp. nov.

**DOI:** 10.1186/s40793-016-0152-z

**Published:** 2016-04-14

**Authors:** Linda Hadjadj, Jaishriram Rathored, Mamadou Bhoye Keita, Caroline Michelle, Anthony Levasseur, Didier Raoult, Pierre-Edouard Fournier, Jean-Marc Rolain, Fadi Bittar

**Affiliations:** Unité de recherche sur les maladies infectieuses et tropicales émergentes (URMITE), UM63, CNRS7278, IRD 198, Inserm 1095, IHU Méditerranée Infection, Faculté de Médecine et de Pharmacie, Aix-Marseille Université, Marseille, France; King Fahad Medical Research Center, King Abdul Aziz University, Jeddah, Saudi Arabia

**Keywords:** *Microbacterium gorillae*, Genome, Culturomics, Taxonomo-genomics, Gorilla stool sample

## Abstract

Strain G3^T^ (CSUR P207 = DSM 26203) was isolated from the fecal sample of a wild gorilla (*Gorilla gorilla* subsp *gorilla*) from Cameroon. It is a Gram-positive, facultative anaerobic short rod. This strain exhibits a 16S rRNA sequence similarity of 98.2 % with *Microbacterium thalassium,* the closest validly published *Microbacterium* species and member of the family *Microbacteriaceae*. Moreover, it shows a low MALDI-TOF-MS score (1.1 to 1.3) that does not allow any identification. Thus, it is likely that this strain represents a new species. Here we describe the phenotypic features of this organism, the complete genome sequence and annotation. The 3,692,770 bp long genome (one chromosome but no plasmid) contains 3,505 protein-coding and 61 RNA genes, including 4 rRNA genes. In addition, digital DNA-DNA hybridization values for the genome of the strain G3^T^ against the closest *Microbacterium* genomes range between 19.7 to 20.5, once again confirming its new status as a new species. On the basis of these polyphasic data, consisting of phenotypic and genomic analyses, we propose the creation of *Microbacterium gorillae* sp. nov*.* that contains the strain G3^T^.

**Electronic supplementary material:**

The online version of this article (doi:10.1186/s40793-016-0152-z) contains supplementary material, which is available to authorized users.

## Introduction

Strain G3^T^ (= CSUR P207 = DSM 26203) is the type strain of *Microbacterium**gorillae* sp. nov. This bacterium is a Gram-positive, non-spore-forming, indole-negative, facultative anaerobic rod shaped bacillus. It was isolated from the feces of western lowland gorilla in Cameroon as part of a culturomics study to describe the bacterial communities of the gorilla gut [1]. By applying a large variety of culture conditions, culturomics allowed previously the isolation of numerous new bacterial species from gorilla fecal samples [1].

Furthermore, since the creation of the genus *Microbacterium* by Orla-Jensenin (1919) [2] to date, 91 bacterial species belonging to this genus have been validly published [3]. These species are Gram-positive and non-endospore-forming bacteria. Many studies have described *Microbactertium* species in diverse origins including human clinical specimens, soil, sea sediments, plants and hairspray [4–7].

In this report, we present a summary classification, phenotypic features for *M. gorillae* sp. nov. strain G3^T^, together with the description of the complete genome sequence and annotation. These characteristics support the circumscription of the species *M. gorillae* [8].

## Organism information

### Classification and features

Information about the fecal sample collection and conservation are described previously [1]. Strain G3^T^ (Table 1) was isolated in January 2012 as part of a culturomics study [1] by cultivation on Columbia agar supplemented with sheep blood (BioMérieux, Craponne, France).

**Table 1:** Classification and general features of *Microbacterium gorillae* strain G3^T^

MIGS ID	Property	Term	Evidence code^a^
	Classification	Domain: *Bacteria*	TAS [34]
		Phylum: *Actinobacteria*	TAS [35]
		Class: *Actinobacteria*	TAS [35]
		Order: *Actinomycetales*	TAS [2]
		Family: *Microbacteriaceae*	TAS [36]
		Genus: *Microbacterium*	TAS [2]
		Species: *Microbacterium gorillae*	IDA
		Type strain: G3^T^	IDA
	Gram stain	Positive	IDA
	Cell shape	Rod-shaped	IDA
	Motility	Non-motile	IDA
	Sporulation	Non-sporulating	IDA
	Temperature range	Mesophilic	IDA
	Optimum temperature	25 °C	IDA
	pH range; Optimum	Not determined	
	Carbon source	Varied (see Additional file 4)	IDA
MIGS-6	Habitat	Gorilla gut	IDA
MIGS-6.3	Salinity	2 % NaCl	IDA
MIGS-22	Oxygen requirement	Facultative anaerobic	IDA
MIGS-15	Biotic relationship	Free living	IDA
MIGS-14	Pathogenicity	Unknown	
	Biosafety level	2	NAS
	Isolation	Gorilla feces	IDA
MIGS-4	Geographic location	Cameroon	IDA
MIGS-5	Sample collection time	July 2011	IDA
MIGS-4.1	Latitude	2° 47′ 2.1768″	IDA
MIGS-4.2	Longitude	13° 1′ 49.6986″	IDA
MIGS-4.4	Altitude	>600 m above sea level	IDA

^a^ Evidence codes - IDA: Inferred from Direct Assay; TAS: Traceable Author Statement

(i.e., a direct report exists in the literature); NAS: Non-traceable Author Statement (i.e., not directly observed for the living, isolated sample, but based on a generally accepted property for the species, or anecdotal evidence). These evidence codes are from the Gene Ontology project [37]. If the evidence is IDA, then the property was directly observed for a live isolate by one of the authors or an expert mentioned in the acknowledgements

**Fig. 1:**
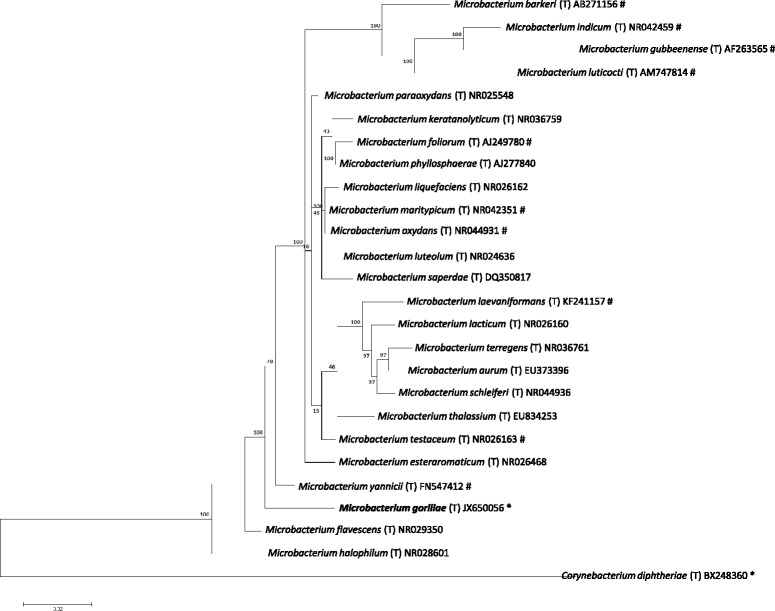
Phylogenetic tree highlighting the position of *Microbacterium gorillae* strain G3^T^ relative to other type strains within the *Microbacterium* genus using 16S rRNA gene. GenBank accession numbers are indicated in parentheses. Sequences were aligned using MUSCLE. Alignments were then cleaned from highly divergent blocks using Gblocks version 0.91b [38]. Maximum likelihood (ML) phylogenetic tree was generated using RAxML [39], employing the GTR GAMMA substitution model with 500 bootstraps. Numbers at the nodes are percentages of bootstrap values obtained by repeating the analysis 500 times to generate a majority consensus tree. *Corynebacterium diphtheriae* was used as outgroup. The scale bar represents a rate of substitution per nucleotide position of 0.02. (T) indicates that the sequence used in the tree is from the type strain of the species.* indicates the strains used in the tree have a sequenced genome. # indicates that a sequenced genome is available for this species but not for the strain used to build the tree

When compared to sequences available in GenBank, the 16S rRNA gene sequence of *M. gorillae* strain G3^T^ (GenBank accession number JX650056) exhibited an identity of 98.2 % with *Microbacterium thalassium*, the closest validly published *Microbacterium* species. This value was equal to the percentage of 16S rRNA gene sequence threshold recommended by Meier-Kolthoff et al. for class *Actinobacteria* to delineate a new species without carrying out DNA-DNA hybridization with maximum error probability of 0.1 % [9]. Figure 1 presents the 16S rRNA based tree for the strain G3^T^ and other *Microbacterium* species.

**Fig. 2:**
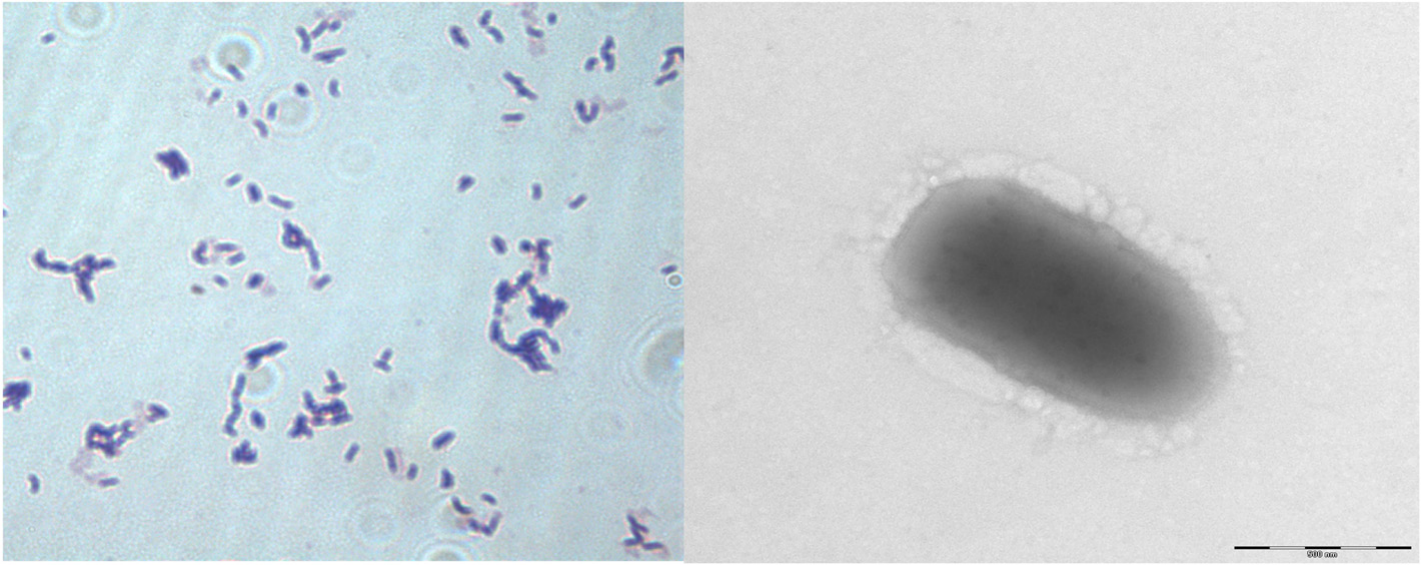
Gram staining (*left panel*) and Transmission electron microscopy using a Morgani 268D (Philips) at an operating voltage of 60 kV (*right panel*) of *M. gorillae* strain G3^T^. The scale bar represents 500 nm

Different growth temperatures (20, 25, 30, 37, 45 °C) were tested. Growth occurred between 25 °C and 37 °C, but the optimal growth was observed at 25 °C, 24 h after inoculation. No growth occurred at 20 and 45 °C. Colonies were 0.8 mm in diameter, appear as gray color on Columbia agar supplemented with sheep blood. Growth of the strain was tested under anaerobic and microaerophilic conditions using GENbag anaer and GENbag microaer systems, respectively (BioMérieux), and under aerobic conditions, with or without 5 % CO_2_. Growth was achieved under aerobic (with and without CO_2_), microaerophilic and anaerobic conditions. Gram staining showed Gram positive short bacilli (Fig. 2, left panel). A motility test with API M medium (BioMérieux) produced a negative result. Cells grown on agar do not sporulate and the rods have a mean length of 1 μm and a mean width of 0.5 μm. Both the length and the diameter were determined by negative staining transmission electron microscopy (Fig. 2, right panel).

Strain G3^T^ exhibited catalase activity but not oxidase activity using ID color catalase and oxidase reagent, respectively (BioMérieux). In assays with API 50CH system (BioMérieux), strain G3^T^ produced acid from esculin, D-cellobiose, D-maltose, D-lactose, D-mannose, D-mannitol, D-saccharose, D-trehalose and gentiobiose. By contrast, acid production was not observed for glycerol, erythritol, D-arabinose, L-arabinose, D-ribose, D-xylose, L-xylose, D-adonitol, methyl-αD-xylopyranoside, D-galactose, D-glucose, L-fructose, L-sorbose, L-rhamnose, dulcitol, inositol, D-sorbitol, methyl-αD-mannopyranoside, Methyl-αD-glucopyranoside, xylitol, D-tagatose, D-turanose, D-lyxose, D-fucose, L-fucose, D-arabitol, L-arabitol, potassium gluconate, potassium 2-cetogluconate, potassium 5-cetogluconate, D-melezitose, D-raffinose, Glycogen, N-acetylglucosamin, amygdalin, arbutin, salicin and hydrolysis of starch. Using APIZYM, positive enzyme activities were observed for esterase (C4), esterase lipase (C8), leucine aramidase, phosphatase acid, naphtol-AS-BI-phosphohydrolase, α-mannosidase, α- glucosidase and N-acetyl-β-glucosaminidase. Negative results for lipase (C14), phosphatase alcalin, valine arylamidase, cystine arylamidase, trypsin, α-chymotrypsin, α-galactosidase, β – galactosidase, β-glucosidase, β-glucuronidase, β-glucosidase, and α-fucosidase.

*M. gorillae* is susceptible to amoxicillin (25 μg), erythromycin (15UI), doxycyclin (30UI), rifampicin (30 μg), vancomycin (50 μg), amoxicillin-clavulanic acid (20 μg + 10 μg), trimethoprim-sulfamethoxazole (1.25 μg / 23.75 μg) and imipenem (10 μg) but resistant to ciprofloxacin (5 μg) and gentamycin (15 μg).

When compared to other *Microbacterium* species [10–16], *M. gorillae* sp. nov. strain G3^T^ exhibited the phenotypic differences detailed in Additional file 1: Table S1.

#### Extended feature descriptions

Matrix-assisted laser-desorption/ionization time-of-flight (MALDI-TOF) MS protein analysis was carried out as previously described [17] using a Microflex spectrometer (Bruker Daltonics, Leipzig, Germany). Twelve distinct deposits were done for strain G3^T^ from 12 isolated colonies. Two microliters of matrix solution (saturated solution of alpha-cyano-4-hydroxycinnamic acid) in 50 % acetronitrile and 2.5 % trifluoroacetic-acid were distributed on each smear and submitted at air drying for five minutes. Then, the spectra from the 12 different colonies were imported into the MALDI BioTyper software (version 2.0, Bruker) and analyzed by standard pattern matching (with default parameter settings) against 5,626 bacterial spectra including 43 spectra from 33 *Microbacterium* species, used as reference data, in the BioTyper database. Briefly, a score ≥ 2 with a species with a validly published name provided allows the identification at the species level, a score ≥ 1.7 but < 2 allows the identification at the genus level; and a score < 1.7 does not allow any identification. For strain G3^T^, no good score was obtained, suggesting that our isolate was not a member of any known species. We incremented our database with the spectrum from strain G3^T^ (Additional file 2: Figure S1). The gel view highlighted spectrum differences with other *Microbacterium* species (Additional file 3: Figure S2).

## Genome sequencing information

### Genome project history

According to phenotypic characteristics of this strain and MALDI-TOF result and because of the low16S rRNA similarity to other members of the genus *Microbacterium**,* it is likely that the strain represents a new species and thus it was chosen for genome sequencing. It was the 20^th^ genome of a *Microbacterium* species (Genomes Online Database) and the first genome of *Microbacterium**gorillae* sp. nov. A summary of the project information is shown in Table 2. The GenBank accession number is CDAR00000000 and consists of 14 contigs. Table 2 shows the project information and its association with MIGS version 2.0 compliance [18].

**Table 2:** Project information

MIGS ID	Property	Term
MIGS-31	Finishing quality	High-quality draft
MIGS-28	Libraries used	Mate pair and paired end
MIGS-29	Sequencing platforms	MiSeq-Illumina
MIGS-31.2	Fold coverage	213X
MIGS-30	Assemblers	Spades
MIGS-32	Gene calling method	Prodigal
	Locus Tag	BN1193
	GenBank ID	CDAR00000000
	GenBank Date of Release	November 04, 2014
	GOLD ID	Gp0025154
	BIOPROJECT	PRJEB7582
MIGS-13	Source Material Identifier	G3^T^
	Project relevance	DSM 26203, CSUR P207

### Growth conditions and genomic DNA preparation

*Microbacterium**gorillae* sp.nov strain G3^T^ (= CSUR P207 = DSM 26203) was grown aerobically on 5 % sheep blood-enriched Columbia agar (BioMérieux) at 25 °C. Bacteria grown on four Petri dishes were resuspended in 3x500μl of TE buffer and stored at 80 °C. Then, 500 μl of this suspension were thawed, centrifuged 3 min at 10,000 rpm and resuspended in 3x100μL of G2 buffer (EZ1 DNA Tissue kit, Qiagen). A first mechanical lysis was performed by glass powder on the Fastprep-24 device (Sample Preparation system, MP Biomedicals, USA) using 2x20 s cycles. DNA was then treated with 2.5 μg/μL lysozyme (30 min at 37 °C) and extracted using the BioRobot EZ1 Advanced XL (Qiagen). The DNA was then concentrated and purified using the Qiamp kit (Qiagen). The yield and the concentration was measured by the Quant-it Picogreen kit (Invitrogen) on the Genios Tecan fluorometer at 50 ng/μl.

### Genome sequencing and assembly

Genomic DNA of *M. gorillae* was sequenced on the MiSeq Technology (Illumina Inc, San Diego, CA, USA) with the 2 applications: paired end and mate paired. The gDNA was barcoded in order to be mixed with 11 others projects with the Nextera Mate Pair sample prep kit (Illumina) and with 17 others projects with the Nextera XT DNA sample prep kit (Illumina).

gDNA was quantified by a Qubit assay with the high sensitivity kit (Life technologies, Carlsbad, CA, USA) to 46.7 ng/μlTo prepare the paired end library, dilution was performed to require 1 ng of each genome as input. The « tagmentation » step fragmented and tagged the DNA. Then limited cycle PCR amplification (12 cycles) completed the tag adapters and introduced dual-index barcodes. After purification on AMPure XP beads (Beckman Coulter Inc, Fullerton, CA, USA), the libraries were then normalized on specific beads according to the Nextera XT protocol (Illumina). Normalized libraries were pooled for sequencing on the MiSeq. The pooled single strand library was loaded onto the reagent cartridge and then onto the instrument along with the flow cell. Automated cluster generation and paired end sequencing with dual index reads were performed in a single 39-h run in 2x250-bp.

Total information of 7.6 Gb was obtained from a 931 K/mm^2^ cluster density with a cluster passing quality control filters of 82.8 % (17,658,000 clusters). Within this run, the index representation for *M. gorillae* was determined to 5.11 %. The 732,922 paired end reads were trimmed and filtered by Trimmomatic tool using the recommended parameters for Illumina sequence data [19].

Two mate pair libraries were prepared with 1 and 1.5 μg of genomic DNA using the Nextera mate pair Illumina guide. The genomic DNA sample was simultaneously fragmented and tagged with a mate pair junction adapter. The pattern of the fragmentation was validated on an Agilent 2100 BioAnalyzer (Agilent Technologies Inc, Santa Clara, CA, USA) with a DNA 7500 labchip. The DNA fragments ranged from 1 kb to 11 kb in size with the majority of fragments at 8.8 and 9.4 kb of size. No size selection was performed and 45 ng for the 1^st^ library and 600 ng for the second library of tagmented fragments were circularized. The circularized DNA was mechanically sheared to small fragments with the majority at 400 and 380 bp on the Covaris device S2 in microtubes (Covaris, Woburn, MA, USA). The library profile was visualized on a High Sensitivity Bioanalyzer LabChip (Agilent Technologies Inc, Santa Clara, CA, USA) and the final concentration library was measured at 0.65 and 0.59 nmol/l respectively. The libraries were normalized at 2nM and pooled. After a denaturation step and dilution at 15 pM, the pool of libraries was loaded onto the reagent cartridge and then onto the instrument along with the flow cell. Automated cluster generation and sequencing run were performed in a single 39-h run in a 2x251-bp. The first libray was loaded three times on a flowcell and the second once. Within these runs, the index representation for *M. gorillae* was determined as an average at 3.51 %. The 1,881,286 paired reads were filtered according to the read qualities. The global paired end and mate pair libraries lead to 2,614,208 paired reads which were trimmed by Trimmomatic [19] then assembled by Spades software using the recommended options “--careful” and “-k 127” to fix the kmer size to 127 [20]. The final assembly identified 14 scaffolds generating a genome size of 3.69 Mb which corresponds to genome coverage of 213X.

### Genome annotation

Open Reading Frames (ORFs) were predicted using Prodigal [21] with default parameters but the predicted ORFs were excluded if they spanned a sequencing gap region. The predicted bacterial protein sequences were searched against the GenBank database [22] and the Clusters of Orthologous Groups (COG) databases using BLASTP. The tRNAScanSE tool [23] was used to find tRNA genes, whereas ribosomal RNAs were found using RNAmmer [24] and BLASTn against the GenBank database. Lipoprotein signal peptides and the number of transmembrane helices were predicted using SignalP [25] and TMHMM [26] respectively. ORFans were identified if their BLASTP E-value was lower than 1e-03 for alignment length greater than 80 amino acids. If alignment lengths were smaller than 80 amino acids, we used an E-value of 1e-05. Such parameter thresholds have already been used in previous works to define ORFans. Artemis [27] was used for data management and DNA Plotter [28] for visualization of genomic features. The Mauve alignment tool (version 2.3.1) was used for multiple genomic sequence alignment [29]. To estimate the mean level of nucleotide sequence similarity at the genome level between *M. gorillae* sp. nov. strain G3T and other members of the genus *Microbacterium*, we used the MAGI home-made software to calculate the average genomic identity of gene sequences (AGIOS) among compared genomes [30]. Briefly, this software combines the Proteinortho software [31] for detecting orthologous proteins in pairwise genomic comparisons, then retrieves the corresponding genes and determines the mean percentage of nucleotide sequence identity among orthologous ORFs using the Needleman-Wunsch global alignment algorithm. Finally, we used Genome-to-Genome Distance Calculator (GGDC) web server available at (http://ggdc.dsmz.de) to estimate of the overall similarity among the compared genomes and to replace the wet-lab DNA-DNA hybridization (DDH) by a digital DDH (dDDH) [32, 33]. GGDC 2.0 BLAST+ was chosen as alignment method and the recommended formula 2 was taken into account to interpret the results.

## Genome properties

**Fig. 3:**
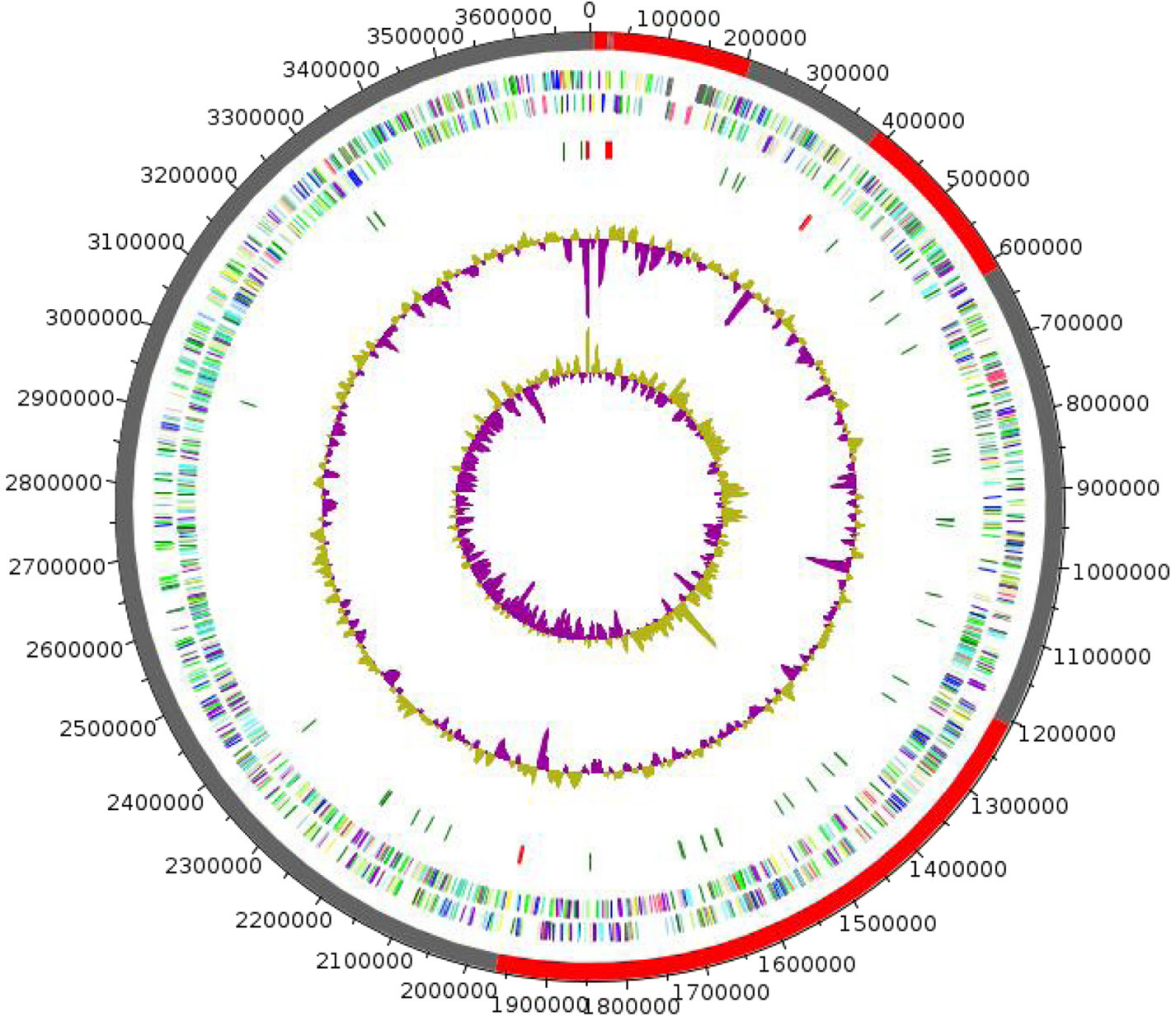
Graphical circular map of the *Microbacterium gorillae* strain G3 ^T^ chromosome. The outer two circles show open reading frames oriented in the forward (colored by COG categories) and reverse (colored by COG categories) directions, respectively. The third circle shows the RNA genes (tRNAs *green*, rRNAs *red*). The fourth circle shows the G + C% content plot. The inner-most circle shows GC skew, *purple* indicating negative values whereas olive for positive values

The genome of *M. gorillae* strain G3^T^ is 3,692,770 bp-long with a 69.3 % G+C content (Table 3, Fig. 3). Of the 3,566 predicted genes, 3,505 were protein-coding genes and 61 were RNA genes, including 4 complete rRNA operons (Additional file 4). A total of 2,412 genes (68.82 %) were assigned a putative function. A total of 6.33 % were identified as Pseudo-genes. The remaining genes were annotated as hypothetical proteins. The properties and the statistics of the genome are summarized in Table 3. The distribution of genes into COGs functional categories is presented in Table 4 and Additional file 4.

**Table 3:** Nucleotide content and gene count levels of the genome

Attribute	Value	% of total^a^
Genome size (bp)	3,692,770	100
DNA coding (bp)	3,396,745	92
DNA G + C (bp)	2,558,287	69.3
DNA scaffolds	14	
Total genes	3,566	100
Protein coding genes	3,505	98.3
RNA genes	61	1.71
Pseudo genes	226	6.33
Genes in internal clusters	ND	ND
Genes with function prediction	2,412	68.8
Genes assigned to COGs	2,202	62.8
Genes with Pfam domains	0	0
Genes with signals peptides	365	10.4
Genes with transmembrane helices	843	24.1
CRISPR repeats	0	0

^a^The total is based on either the size of the genome in base pairs or the total number of protein coding genes in the annotated genome

ND: Not determined

**Table 4:** Number of genes associated with the 25 general COG functional categories

Code	Value	% of total^a^	Description
J	149	4.25	Translation
A	1	0.03	RNA processing and modification
K	269	7.67	Transcription
L	109	3.11	Replication, recombination and repair
B	0	0.00	Chromatin structure and dynamics
D	16	0.46	Cell cycle control, mitosis and meiosis
Y	0	0.00	Nuclear structure
V	41	1.17	Defense mechanisms
T	75	2.14	Signal transduction mechanisms
M	82	2.34	Cell wall/membrane biogenesis
N	1	0.03	Cell motility
Z	0	0.00	Cytoskeleton
W	0	0.00	Extracellular structures
U	24	0.68	Intracellular trafficking and secretion
O	66	1.88	Posttranslational modification, protein turnover, chaperones
C	150	4.28	Energy production and conversion
G	257	7.33	Carbohydrate transport and metabolism
E	325	9.27	Amino acid transport and metabolism
F	69	1.97	Nucleotide transport and metabolism
H	83	2.37	Coenzyme transport and metabolism
I	151	4.31	Lipid transport and metabolism
P	184	5.25	Inorganic ion transport and metabolism
Q	95	2.71	Secondary metabolites biosynthesis, transport and catabolism
R	410	11.70	General function prediction only
S	145	4.14	Function unknown
-	1303	37.17	Not in COGs

^a^ The total is based on the total number of protein coding genes in the annotated genome

## Insights from the genome sequence

Here, we compared the genome sequences of *M. gorillae* strain G3^T^ (CDAR00000000) with those of *Microbacterium barkeri* strain 2011-R4 (AKVP00000000), *Microbacterium maritypicum* strain MF109 (ATAO00000000), *Microbacterium indicum* strain DSM 19969 (AULR00000000), *Microbacterium laevaniformans* strain OR221 (AJGR00000000), *Microbacterium luticocti* strain DSM 19459 (AULS00000000), *Microbacterium paraoxydans* strain 77MFTsu3.2 (AQYI00000000), *Microbacterium testaceum* strain StLB037 (AP012052) and *Microbacterium yannicii* strain PS01 (CAJF00000000). The draft genome of *M. gorillae* has a larger size than those of *M. indicum**,**M. luticocti**,**M. laevaniformans**,**M. paraoxydans* and *M. barkeri**,* (3.69 vs 2.81, 3.11, 3.43, 3.48 and 3.64 Mb respectively) but is smaller than those of *M. maritypicum**,**M. testaceum* and *M. yannicii* (3.69 vs 4.0, 3.98 and 3.95 Mb respectively). The G+C content of *M. gorillae* is higher than those of *M. laevaniformans* and *M. maritypicum* (69.3 vs 68.0 and 68.2 % respectively) but lower than those of *M. indicum**,**M. luticocti**,**M. testaceum**,**M. yannicii**,**M. paraoxydans* and *M. barkeri* (69.3 vs 71.4, 70.7, 70.3, 69.5, 69.5, 69.2 %, respectively). The gene content of *M. gorillae* is lower than those of *M. maritypicum* and *M. testaceum*, (3,505 vs 3,856 and 3,676 genes respectively) but higher than those of*, M. paraoxydens,**M. yannicii**,**M. laevaniformans**,**M. barkeri**,**M. luticocti* and *M. indicum* (3,312, 3,279. 3,249, 3,099, 2,355, 2,183 genes respectively) (Table 5). However the distribution of genes into COG categories was similar in all compared genomes (Additional file 5: Figure S3). In addition, *M. gorillae* shares 1,593, 1,658, 1,269, 1,396, 1,390, 1,416, 1,498 and 1,497 orthologous genes with *M. barkeri*, *M. maritypicum*, *M. indicum*, *M. laevaniformans*, *M. luticocti*, *M. paraoxydans*, *M. testaceum* and *M. yannici**i* respectively (Table 5). Among compared genomes except *M. gorillae*, AGIOS values range from 75.51 % between *M. indicum* and *M. maritypicum* to 85.33 % between *M. maritypicum* and *M. barkeri* . When *M. gorillae* was compared to other species, AGIOS values range from 75.22 % with *M. maritypicum* to 76.41 % with *M. luticocti* (Table 5). dDDH estimation of the strain G3^T^ against the compared genomes ranged between 19.70 to 20.50. These values are very low and below the cutoff of 70 %, thus confirming again the new species status of the strain G3^T^.

**Table 5:** Genomic comparison of *M. gorillae* sp. nov., strain G3^T^ with other *Microbacterium* species.

Species	*M. gorillae*	*M. barkeri*	*M. maritypicum*	*M. indicum*	*M. laevaniformans*	*M. luticocti*	*M. paraoxydans*	*M. testaceum*	*M. yannicii*
*M. gorillae*	**3,505**	1,593	1,658	1,269	1,396	1,390	1,416	1,498	1,497
*M. barkeri*	75.91	**3,099**	2,111	1,390	1,511	1,461	1,595	1,685	1,684
*M. maritypicum*	75.22	85.33	**3,856**	1,429	1,581	1,549	1,634	1,755	1,734
*M. indicum*	75.39	76.16	75.51	**2,183**	1,296	1,191	1,324	1446	1,349
*M. laevaniformans*	75.80	76.59	76.07	76.05	**3,249**	1414	1,602	1,638	1,580
*M. luticocti*	76.41	76.99	76.50	76.34	77.94	**2,355**	1,395	1,433	1,512
*M. paraoxydans*	75.66	76.36	75.90	76.43	78.49	77.34	**3,312**	1,710	1,632
*M. testaceum*	75.64	76.48	75.84	76.30	77.64	77.64	77.52	**3,676**	1,723
*M. yannicii*	75.85	76.89	76.34	76.53	78.06	78.60	77.82	78.10	**3,279**

The numbers of orthologous proteins shared between genomes (upper right triangle), average percentage similarity of nucleotides corresponding to orthologous protein shared between genomes (lower left triangle) and numbers of proteins per genome (bold)

## Conclusions

On the basis of phenotypic characteristics, phylogenetic position, genomic analyses (taxonogenomics) and GGDC results, we formally propose the creation of *Microbacterium**gorillae* sp. nov*.* that contains the strain G3^T^. This strain has been isolated from a gorilla stool sample collected from Cameroon.

## Taxonomic and nomenclatural proposals

### Description of *Microbacterium**gorillae* sp. nov.

*Microbacterium**gorillae* (go.ril’lae. NL neut. gen gorilla, pertaining to a gorilla from which the stool sample was obtained).

Cells stain Gram-positive, are small rod, non-endospore-forming, non-motile and have a diameter of 0.5 μm and a length of 1 μm. Colonies are gray and 2 mm in diameter on blood-enriched Columbia agar. Growth occurs between 25 and 37 °C, with optimal growth observed at 25 °C.

Strain G3^T^ exhibited catalase activity but not oxidase activity. Strain produces acid from esculin, D-cellobiose, D-maltose, D-lactose, D-mannose, D-mannitol, D-saccharose, D-trehalose and gentiobiose but not from glycerol, erythritol, D-arabinose, L-arabinose, D-ribose, D-xylose, L-xylose, D-adonitol, methyl-αD-xylopyranoside, D-galactose, D-glucose, L-fructose, L-sorbose, L-rhamnose, dulcitol, inositol, D-sorbitol, methyl-αD-mannopyranoside, Methyl-αD-glucopyranoside, xylitol, D-tagatose, D-turanose, D-lyxose, D-fucose, L-fucose, D-arabitol, L-arabitol, potassium gluconate, potassium 2-cetogluconate, potassium 5-cetogluconate, D-melezitose, D-raffinose, Glycogen, N-acetylglucosamin, amygdalin, arbutin, salicin and hydrolysis of starch.

Positive enzyme activities were observed for esterase (C4), esterase lipase (C8), leucine aramidase, phosphatase acid, naphtol-AS-BI-phosphohydrolase, α-mannosidase, α- glucosidase and N-acetyl-β-glucosaminidase. Negative results for lipase (C14), phosphatase alcalin, valine arylamidase, cystine arylamidase, trypsin, α-chymotrypsin, α-galactosidase, β – galactosidase, β-glucosidase, β-glucuronidase, β-glucosidase, and α-fucosidase.

*M. gorillae* is susceptible to amoxicillin, erythromycin, doxycyclin, rifampicin, vancomycin, amoxicillin-clavulanic acid, trimethoprim-sulfamethoxazole and imipenem but resistant to ciprofloxacin and gentamycin.

The G+C content of the genome is 69.3 %. The 16S rRNA and genome sequences are deposited in GenBank under accession numbers JX650056 and CDAR00000000, respectively. The type strain G3^T^ (= CSUR P207 = DSM 26203) was isolated from the fecal sample of a western lowland gorilla from Cameroon.

## Additional files

Additional file 1: Table S1. Differential phenotypic characteristics between *Microbacterium gorillae* sp. nov. strain G3^T^ and others *Microbacterium* strains. (DOCX 14 kb) Additional file 2: Figure S1. Reference mass spectrum from *M. gorillae* strain G3^T^. Spectra from 12 individual colonies were compared and a reference spectrum was generated. (PPTX 44 kb) Additional file 3: Figure S2. Gel view comparing *Microbacterium gorillae* strain G3^T^ spectra with other members of the genus *Microbacterium*. The gel view displays the raw spectra of all loaded spectrum files arranged in a pseudo-gel like look. The x-axis records the m/z value. The left y-axis displays the running spectrum number originating from subsequent spectra loading. The peak intensity is expressed by a gray-scale scheme code. The color bar and the right y-axis indicate the relation between the color a peak is displayed with and the peak intensity in arbitrary units. Displayed species are indicated on the right. (PPTX 76 kb) Additional file 4: Folder S1. Annotation results. (RAR 1566 kb) Additional file 5: Figure S3. Distribution of functional classes of predicted genes of *M. gorillae* strain G3^T^ with 8 members of *Microbacterium* genus. (PPTX 63 kb)

## Abbreviations

CSUR: Collection de souches de l’Unité des Rickettsies
URMITE: Unité de Recherche sur les Maladies Infectieuses et Tropicales Emergentes
DSM: Deutsche Sammlung von Mikroorganismen
MALDI-TOF MS: Matrix-assisted laser-desorption/ionization time-of-flight mass spectrometry
TE buffer: Tris-EDTA buffer
GGDC: Genome-to-Genome Distance Calculator
dDDH: digital DNA-DNA hybridization
