# Supplementary material for: Non contiguous-finished genome sequence and description of Microbacterium gorillae sp. nov
Source: Stand Genomic Sci. 2016 Apr 14;11:32. doi: 10.1186/s40793-016-0152-z (PMC4832456; doi:10.1186/s40793-016-0152-z)
Supplement: Additional file 1: Table S1. — Differential phenotypic characteristics between Microbacterium gorillae sp. nov. strain G3T and others Microbacterium strains. (DOCX 14 kb) [file 40793_2016_152_MOESM1_ESM.docx]

**Table S1.** Differential phenotypic characteristics between *Microbacterium gorillae* sp. nov. strain G3^T^ and others *Microbacterium* strains.

| **Characteristic** | **1** | **2** | **3** | **4** | **5** | **6** | **7** | **8** | **9** |
| --- | --- | --- | --- | --- | --- | --- | --- | --- | --- |
| Gram stain | + | + | + | + | + | + | + | + | + |
| Salt tolerance | ≤2% | - | <2% | 8% | 6.5% | na | ≤5% | na | - |
| Motility | - | + | na | + | + | + | na | - | na |
| **Production of** |  |  |  |  |  |  |  |  |  |
| Catalase | + | + | na | - | na | + | na | + | - |
| Oxidase | - | na | na | na | na | - | na | - | na |
| Urease | - | - | na | + | na | na | na | na | - |
| Indole | - | na | na | - | na | na | na | na | + |
| Gelatin hydrolysis | - | + | + | + | + | + | - | + | na |
| **Acid production from** |  |  |  |  |  |  |  |  |  |
| L-arabinose | - | + | na | - | - | + | - | na | + |
| D-ribose | - | - | - | na | - | + | - | + | + |
| D-xylose | - | + | na | na | - | na | - | + | + |
| L-xylose | - | - | na | na | na | na | na | na | + |
| D-galactose | - | - | na | w | - | + | + | + | - |
| D-glucose | - | - | na | - | + | + | + | na | + |
| L-rhamnose | - | + | - | + | - | + | - | - | - |
| Inositol | - | - | na | na | na | na | na | na | + |
| Mannitol | + | - | na | na | + | + | + | na | + |
| D-raffinose | - | - | na | na | - | - | - | na | + |
| Starch | - | + | na | w | - | na | + | na | - |
| **Habitat** | Gorilla gut | Raw domestic sewage | Crops and Plants | Sewage sludge compost | Sea water, marine mud | Patients | Sewage | Plants, CF patients | Deep-sea  sediment |

+: positive result, -: negative result, na: data not available, w: weak positive

1**:** *M. gorillae*, 2 : *M. barkeri*, 3 : *M. testaceum*, 4 : *M. luticocti*, 5 : *M. maritypicum*, 6*: M. paraoxydans,* 7*: M. levaniformans,* 8*: M. yannicii,* 9 *: M. indicum.*
