# Supplementary figures and images for: Non contiguous-finished genome sequence and description of Microbacterium gorillae sp. nov
Source: Stand Genomic Sci. 2016 Apr 14;11:32. doi: 10.1186/s40793-016-0152-z (PMC4832456; doi:10.1186/s40793-016-0152-z)

## Slide 1
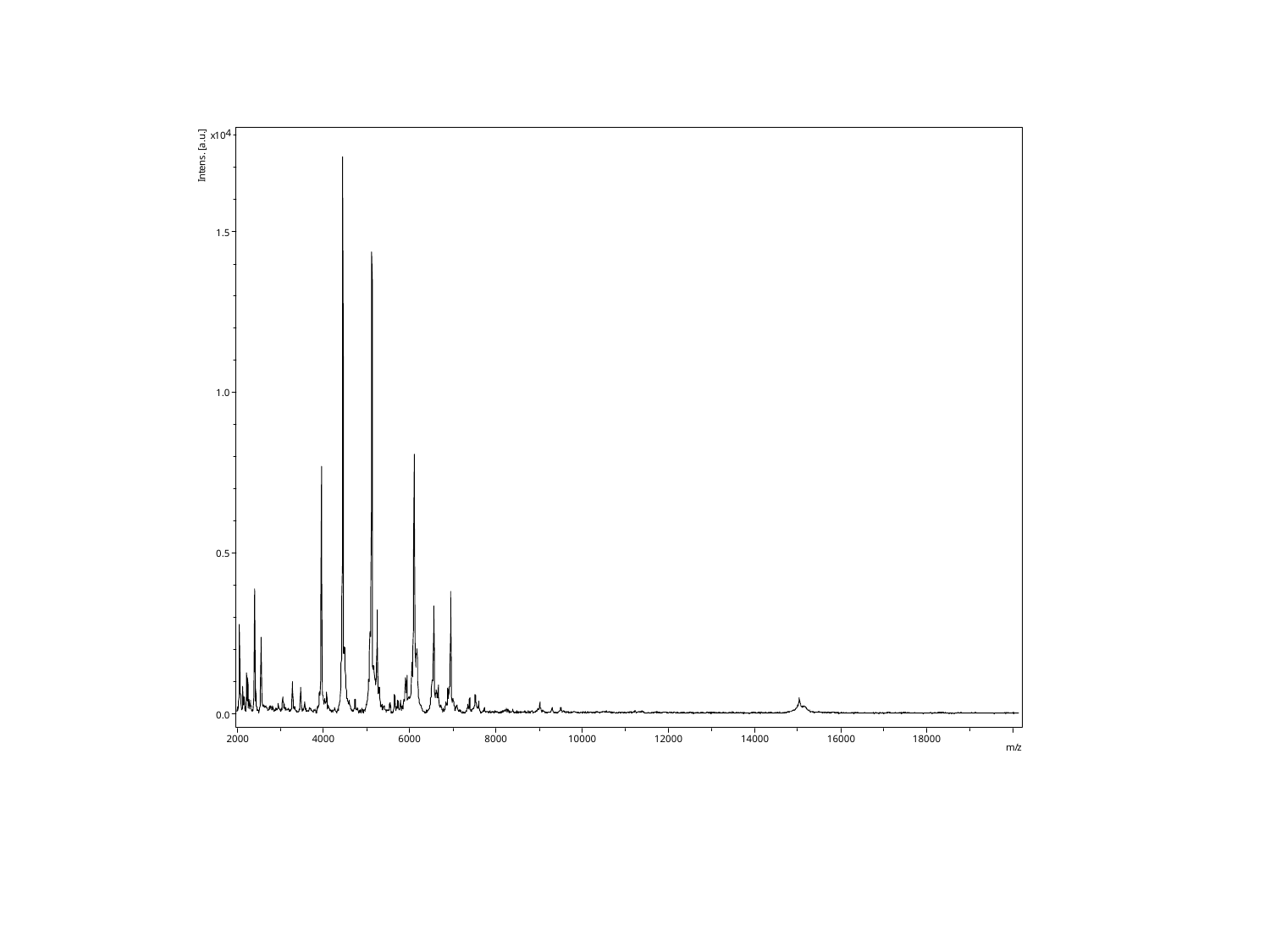

Supplement: Additional file 2: Figure S1. — Reference mass spectrum from M. gorillae strain G3T. Spectra from 12 individual colonies were compared and a reference spectrum was generated. (PPTX 44 kb) [file 40793_2016_152_MOESM2_ESM.pptx]
