# Supplementary material for: Non contiguous-finished genome sequence and description of Microbacterium gorillae sp. nov
Source: Stand Genomic Sci. 2016 Apr 14;11:32. doi: 10.1186/s40793-016-0152-z (PMC4832456; doi:10.1186/s40793-016-0152-z)
Supplement: Additional file 3: Figure S2. — Gel view comparing Microbacterium gorillae strain G3T spectra with other members of the genus Microbacterium. The gel view displays the raw spectra of all loaded spectrum files arranged in a pseudo-gel like look. The x-axis records the m/z value. The left y-axis displays the running spectrum number originating from subsequent spectra loading. The peak intensity is expressed by a gray-scale scheme code. The color bar and the right y-axis indicate the relation between the color a peak is displayed with and the peak intensity in arbitrary units. Displayed species are indicated on the right. (PPTX 76 kb) [file 40793_2016_152_MOESM3_ESM.pptx]

## Slide 1
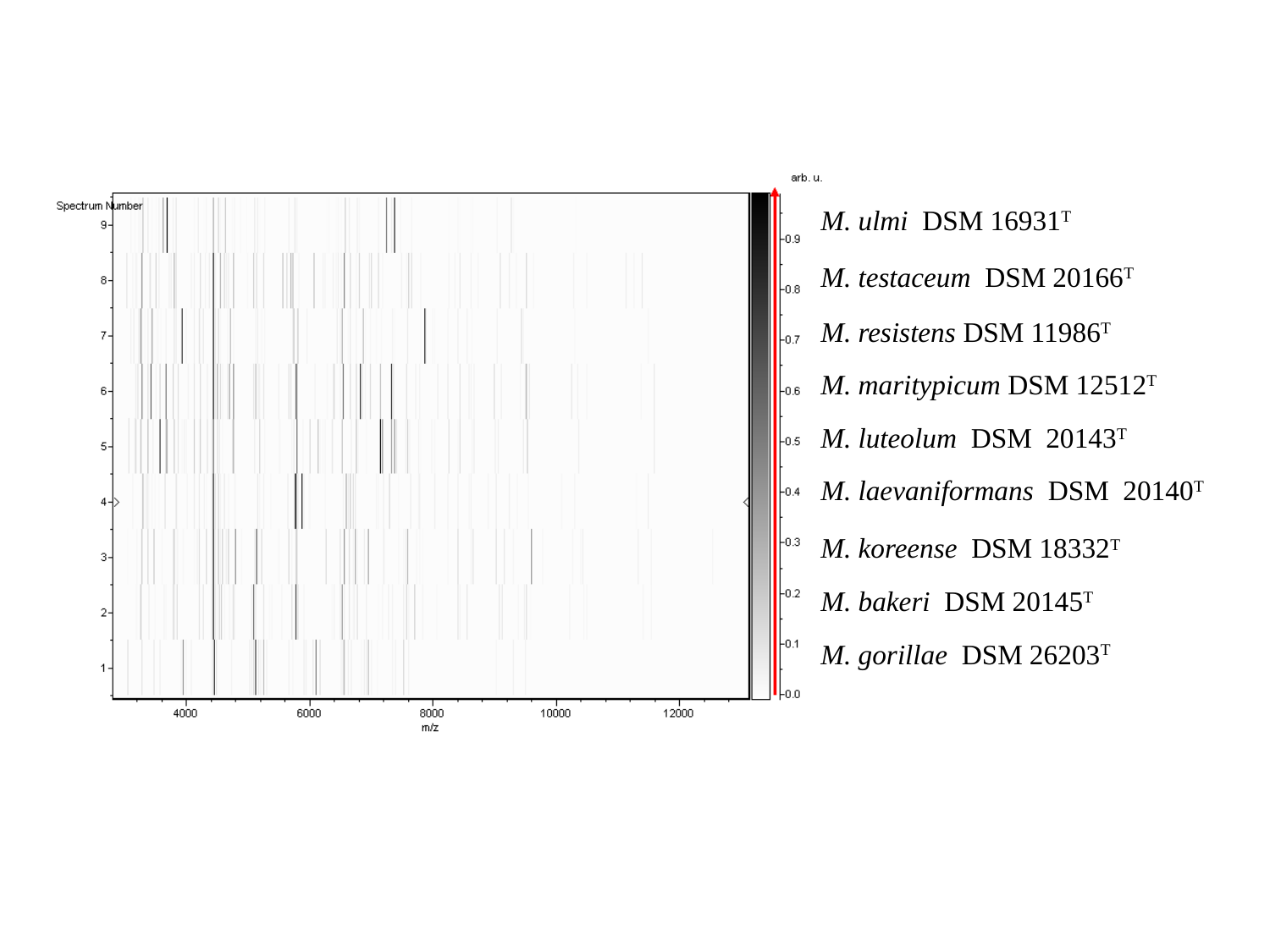

M. ulmi DSM 16931T
M. testaceum DSM 20166T
M. resistens DSM 11986T
M. maritypicum DSM 12512T
M. luteolum DSM 20143T
M. laevaniformans DSM 20140T
M. koreense DSM 18332T
M. bakeri DSM 20145T
M. gorillae DSM 26203T
