# Supplementary material for: Non contiguous-finished genome sequence and description of Microbacterium gorillae sp. nov
Source: Stand Genomic Sci. 2016 Apr 14;11:32. doi: 10.1186/s40793-016-0152-z (PMC4832456; doi:10.1186/s40793-016-0152-z)
Supplement: Additional file 5: Figure S3. — Distribution of functional classes of predicted genes of M. gorillae strain G3T with 8 members of Microbacterium genus. (PPTX 63 kb) [file 40793_2016_152_MOESM5_ESM.pptx]

## Slide 1
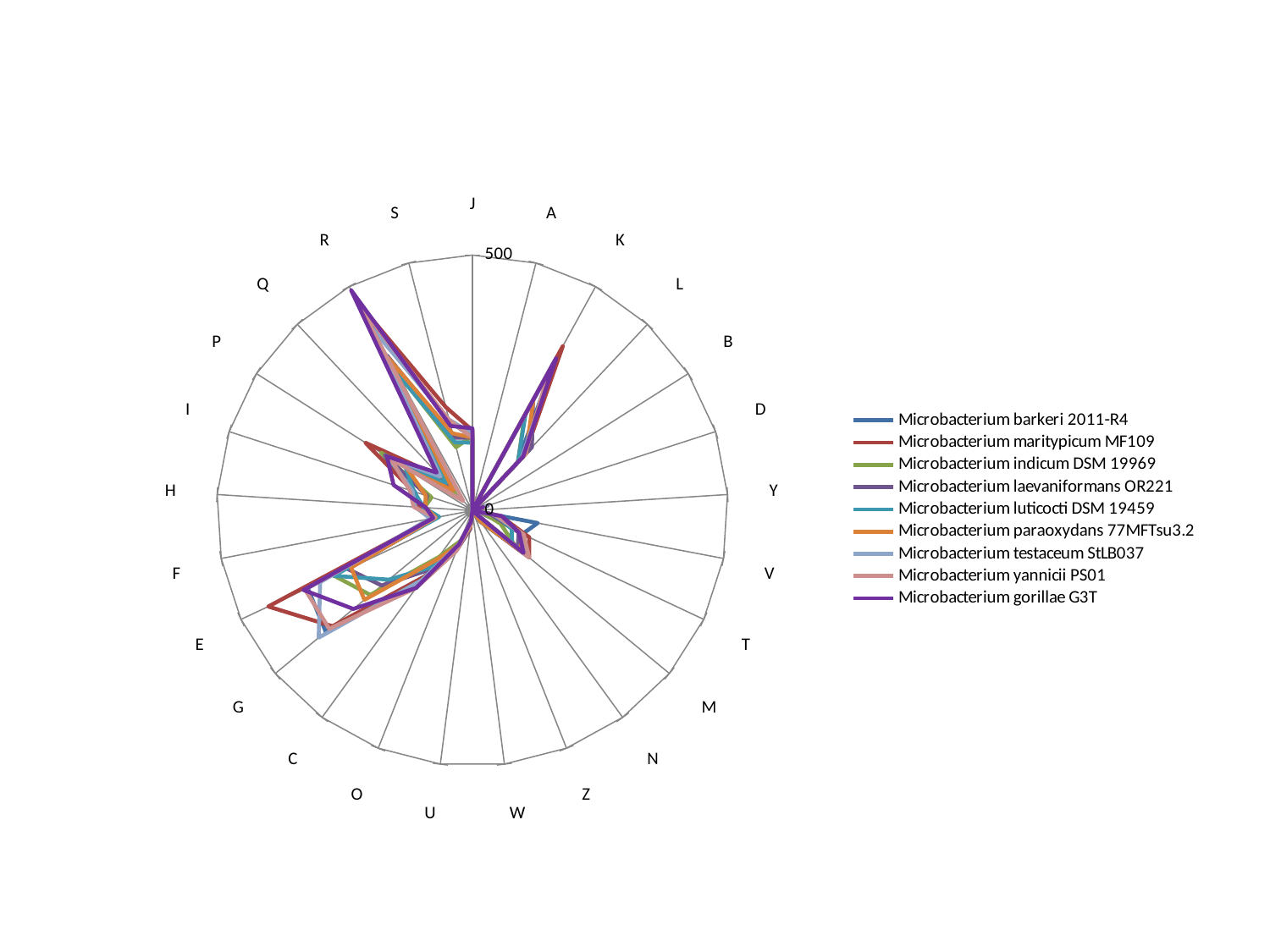

### Chart
| Category | Microbacterium barkeri 2011-R4 | Microbacterium maritypicum MF109 | Microbacterium indicum DSM 19969 | Microbacterium laevaniformans OR221 | Microbacterium luticocti DSM 19459 | Microbacterium paraoxydans 77MFTsu3.2 | Microbacterium testaceum StLB037 | Microbacterium yannicii PS01 | Microbacterium gorillae G3T |
|---|---|---|---|---|---|---|---|---|---|
| J | 144.0 | 157.0 | 146.0 | 143.0 | 133.0 | 143.0 | 150.0 | 143.0 | 161.0 |
| A | 1.0 | 2.0 | 1.0 | 1.0 | 1.0 | 2.0 | 1.0 | 1.0 | 1.0 |
| K | 334.0 | 367.0 | 212.0 | 246.0 | 216.0 | 248.0 | 334.0 | 310.0 | 340.0 |
| L | 129.0 | 149.0 | 130.0 | 170.0 | 130.0 | 153.0 | 141.0 | 150.0 | 145.0 |
| B | 0.0 | 0.0 | 0.0 | 0.0 | 0.0 | 0.0 | 0.0 | 0.0 | 0.0 |
| D | 23.0 | 24.0 | 20.0 | 31.0 | 24.0 | 23.0 | 28.0 | 23.0 | 22.0 |
| Y | 0.0 | 0.0 | 0.0 | 0.0 | 0.0 | 0.0 | 0.0 | 0.0 | 0.0 |
| V | 130.0 | 56.0 | 22.0 | 36.0 | 49.0 | 46.0 | 43.0 | 47.0 | 59.0 |
| T | 108.0 | 123.0 | 61.0 | 100.0 | 85.0 | 111.0 | 111.0 | 111.0 | 101.0 |
| M | 133.0 | 144.0 | 109.0 | 115.0 | 101.0 | 120.0 | 139.0 | 144.0 | 130.0 |
| N | 27.0 | 24.0 | 1.0 | 2.0 | 27.0 | 26.0 | 0.0 | 2.0 | 2.0 |
| Z | 0.0 | 0.0 | 0.0 | 0.0 | 0.0 | 0.0 | 3.0 | 0.0 | 0.0 |
| W | 0.0 | 0.0 | 0.0 | 0.0 | 0.0 | 0.0 | 0.0 | 0.0 | 0.0 |
| U | 35.0 | 37.0 | 24.0 | 36.0 | 27.0 | 32.0 | 21.0 | 28.0 | 22.0 |
| O | 75.0 | 83.0 | 64.0 | 82.0 | 78.0 | 72.0 | 71.0 | 85.0 | 71.0 |
| C | 160.0 | 160.0 | 113.0 | 143.0 | 137.0 | 119.0 | 168.0 | 187.0 | 187.0 |
| G | 372.0 | 355.0 | 259.0 | 230.0 | 212.0 | 274.0 | 390.0 | 363.0 | 302.0 |
| E | 356.0 | 441.0 | 299.0 | 269.0 | 300.0 | 262.0 | 329.0 | 362.0 | 365.0 |
| F | 73.0 | 76.0 | 84.0 | 71.0 | 66.0 | 73.0 | 84.0 | 74.0 | 78.0 |
| H | 102.0 | 104.0 | 94.0 | 95.0 | 95.0 | 92.0 | 112.0 | 115.0 | 91.0 |
| I | 118.0 | 121.0 | 84.0 | 93.0 | 120.0 | 96.0 | 123.0 | 129.0 | 162.0 |
| P | 209.0 | 248.0 | 211.0 | 203.0 | 154.0 | 149.0 | 194.0 | 189.0 | 198.0 |
| Q | 77.0 | 90.0 | 47.0 | 59.0 | 74.0 | 54.0 | 91.0 | 26.0 | 102.0 |
| R | 420.0 | 466.0 | 318.0 | 346.0 | 308.0 | 340.0 | 406.0 | 459.0 | 492.0 |
| S | 180.0 | 209.0 | 128.0 | 147.0 | 138.0 | 156.0 | 184.0 | 183.0 | 171.0 |
